# Different clinical presentation of intralabyrinthine schwannomas – a systematic review^☆^

**DOI:** 10.1016/j.bjorl.2018.05.007

**Published:** 2018-06-21

**Authors:** Thaís Gomes Abrahão Elias, Adriana Perez Neto, Ana Tereza Silveira Zica, Marcos Luiz Antunes, Norma de Oliveira Penido

**Affiliations:** Universidade Federal de São Paulo (UNIFESP), Departamento de Otorrinolaringologia e Cirurgia de Cabeça e Pescoço, São Paulo, SP, Brazil

**Keywords:** Schwannoma, Neuroma, Neurilemmoma, Intralabyrinthine schwannoma, Schwannoma, Neuroma, Neurilemoma, Schwannoma intralabiríntico

## Abstract

**Introduction:**

Intralabyrinthine schwannoma is a rare, benign tumor that affects the most terminal portions of the vestibular and cochlear nerves. This tumor can be classified into 10 subtypes, according to its inner ear location.

**Objective:**

To carry out a comprehensive review of the most frequent auditory manifestations secondary to the intralabyrinthine schwannoma, describing the possible underlying pathophysiological mechanisms.

**Methods:**

Systematic review of the literature until October 2017 using the PubMed, Web of Science and Scopus databases. The inclusion criteria were clinical manifestations of the intralabyrinthine schwannoma. Three researchers independently assessed the articles and extracted relevant information. The description of a case of an intravestibular subtype intralabyrinthine schwannoma with multiple forms of clinical presentations was used as an example.

**Results:**

Twenty-seven studies met our inclusion criteria. The most common intralabyrinthine schwannoma subtype was the intracochlear, followed by the intravestibular type. All the cases demonstrated hearing loss, usually progressive hearing loss.

**Conclusion:**

The diagnosis of intralabyrinthine schwannomas is based on high-resolution magnetic resonance imaging and should be included in the differential diagnosis of patients with vestibulocochlear complaints. Although there are approximately 600 cases in the literature, we still lack a detailed description of the clinical evolution of the patients, correlating it with MRI findings of temporal bones and tumor subtype.

## Resumo

**Introdução:**

Schwannoma intralabiríntico é um tumor benigno, raro, que afeta as porções mais terminais dos nervos vestibular e coclear. Este tumor pode ser classificado, de acordo com sua localização na orelha interna, em 10 subtipos.

**Objetivo:**

Realizar uma revisão abrangente das manifestações auditivas mais frequentes secundárias ao schwannoma intralabiríntico e descrever os possíveis mecanismos fisiopatológicos subjacentes.

**Método:**

Revisão sistemática da literatura até outubro de 2017 nas bases de dados *PubMed*, *Web of Science* e *Scopus*. O critério de inclusão foi manifestações clínicas do schwannoma intralabiríntico. Três pesquisadores avaliaram de forma independente os artigos e extraíram informações relevantes. Exemplificamos com a descrição de um caso de schwannoma intralabiríntico subtipo intravestibular com múltiplas formas de apresentações clínicas.

**Resultados:**

Vinte sete estudos contemplaram nossos critérios de inclusão. O subtipo do schwannoma intralabiríntico mais comum encontrado foi o intracoclear, seguido pelo intravestibular. Todos os casos apresentaram alteração auditiva, normalmente perda auditiva progressiva.

**Conclusão:**

O diagnóstico de schwannomas intralabirínticos baseia-se em exames de ressonância magnética de alta resolução e deve ser incluído no diagnóstico diferencial de pacientes com queixas vestibulococleares. Apesar de termos aproximadamente 600 casos na literatura, ainda nos falta descrição detalhada da evolução clínica dos pacientes em correlação com achados na ressonância magnética de ossos temporais e o subtipo tumoral.

## Introduction

Intralabyrinthine schwannoma is a rare, benign tumor that affects the most terminal portions of the vestibular and cochlear nerves. It can be located in the vestibule, cochlea or semicircular canals.^1^ Patients usually have nonspecific symptoms, including hearing loss, tinnitus and vertigo.^2^ Among the resulting symptoms, the most frequent is hearing loss, which affects 95% of the patients. Most times, this loss is slow and progressive, but it may be sudden or fluctuating. Less common symptoms include tinnitus (51%), imbalance (35%), vertigo (22%) and ear fullness (2%), which may be present alone or in combination.^3^, ^4^

In 1972, Karlan et al.^5^ reported the first intraoperative findings of the intralabyrinthine schwannoma, which were complemented in 1979 with the histopathological description of a patient's temporal bone with the same lesion.^6^ However, only after 1987 studies were published emphasizing the importance of magnetic resonance imaging (MRI) studies in the diagnosis of this disease.^7^ Currently, the gold-standard examination for the diagnosis of intralabyrinthine schwannoma is the temporal bone MRI, which may show tumor enhancement in post-gadolinium T1-weighted images and filling defects in T2-weighted images.^5^, ^6^

The intralabyrinthine schwannoma can be classified according to its location in the inner ear. Initially, seven subtypes were identified by Kennedy et al., and subsequently, in 2013, Van Abel et al., aiming at using a more specific nomenclature regarding tumor location, added another three subtypes (Table 1).^7^, ^8^ The controversy remains regarding which subtype of the intralabyrinthine schwannoma would be the most common; however, most studies state that the intracochlear location is the most often found, with semicircular canals being more rarely involved.^9^

**Table 1:** Modified Kennedy classification system.^7^

Schwannoma subtypes	Location
Intravestibular	Vestibule and/or SCC
Intracochlear	Cochlea
Intravestibulocochlear	Vestibule and/or SCC + cochlea
Transmodiolar	Cochlea + IAM
Transmacular	Vestibule and/or SCC + IAM
Transotic	Vestibule and/or SCC + Cochlea + IAM +
Middle ear	
Tympanolabyrinthine	Vestibule and/or SCC + cochlea + Middle ear
Translabyrinthine	Vestibule and/or SCC + cochlea + IAM
Affecting the CPA	CPA ± Cochlea ± vestibule and/or SCC ± IAM ± Middle ear
Unspecified	± Cochlea ± vestibule and/or SCC

SCC, semicircular canals; IAM, internal auditory meatus; CPA, cerebellopontine angle.

To the best of our knowledge, no previous study has stringently assessed the characteristics of hearing loss secondary to intralabyrinthine schwannoma and its association with the lesion subtype. Thus, the aim of this study is to carry out a comprehensive review of the most frequent hearing manifestations secondary to the intralabyrinthine schwannoma, describing the possible underlying pathophysiological mechanisms. At the same time, we report the case of a patient with hearing loss secondary to the intralabyrinthine schwannoma of the intravestibular subtype.

## Methods

### Systematic review

A systematic review was carried out in the Pubmed, Web of Science and Scopus databases until October 2017. The following descriptors were used, indicated in the Portuguese language Health Sciences Descriptors (DeCS) terminology (Schwannoma, Neurilemoma, Neuroma), as well as in the English language (Schwannoma, Neurilemmoma, Neuroma). The search strategies for the different databases are shown in Table 2.

**Table 2:** Search strategy.

Database	Search strategy	Articles
Pubmed	(Neurilemmoma or Neuroma or Schwannoma) and (Intralabyrinthine)	100
Web of Science	(Neurilemmoma or Neuroma or Schwannoma) and (Intralabyrinthine)	113
Scopus	(Neurilemmoma or Neuroma or Schwannoma) and (Intralabyrinthine)	117

The inclusion criteria consisted of studies describing clinical manifestations of intralabyrinthine schwannoma, irrespective of its subtype. Initially, the title and abstract in English were read, and then the full-text of studies that met the inclusion criteria were read in their entirety.

Three investigators independently assessed the articles and extracted relevant information, and discrepancies were resolved by mutual agreement. In a complementary manner, we exemplified the literature review findings by describing a clinical case of an intralabyrinthine schwannoma (intravestibular subtype) with multiple forms of clinical presentation.

## Results

### Description of the systematized literature searches

**Figure 1:**
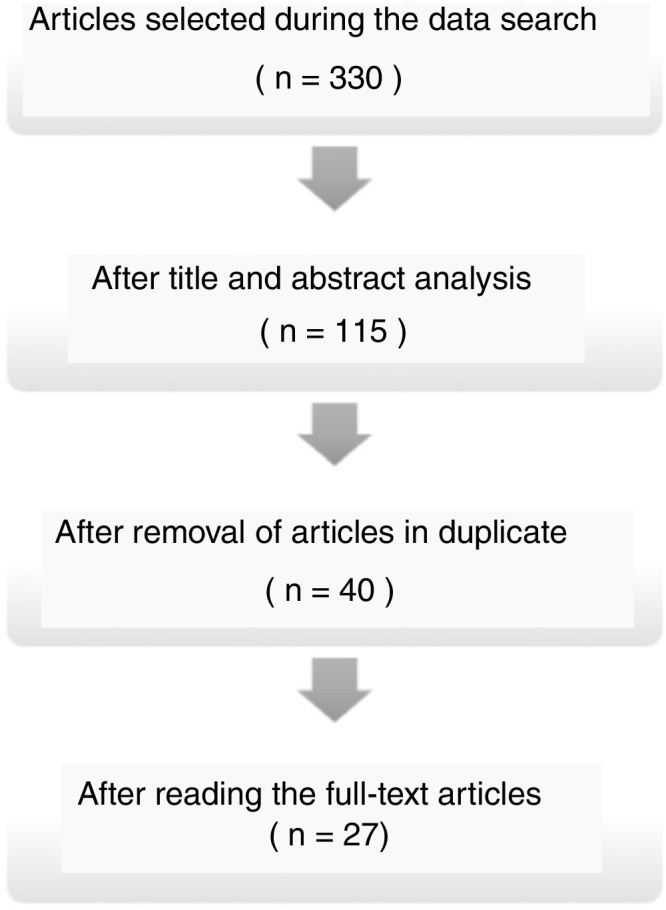
Flowchart showing the article selection process.

The search for articles in the databases identified 330 articles, of which 27 were included in this review (Fig. 1).

According to the Oxford Center for Evidence-Based Medicine classification about the levels of scientific evidence of the 27 articles read as full-text articles, 5 were Systematic Reviews (with homogeneity) of Cohort Studies (level 2A), 1 was an Article on Observation of Therapeutic Results, Ecological Study (level 2C) and 21 were Case Reports (level 4).

Regarding the main tumor location, there was a consensus among the studies that the intracochlear subtype in all studies was the most frequent, followed by the intravestibular type, except the one by Salzman et al., who found the transmodiolar subtype as the second most frequent type (Table 3).

**Table 3:** Main intralabyrinthine schwannomas subtypes.

Author	Year	*n*	Location
Gosselin et al.	2015	66	50.9% intracochlear
			38.2% intravestibular
			10.9% intravestibulocochlear
Dubernard et al.	2014	110	50% intracochlear
			19.2% intravestibular
			14.5% transmodiolar
			11.8% intravestibulocochlear
			2.7% transmacular
			1.8% tympanolabyrinthine
Van Abel et al.	2013	234	51% intracochlear
			29% intravestibular
			9% intravestibulocochlear
			5% transmodiolar
			1% transmacular
			1% translabyrinthine
Salzman et al.	2012	45	31.11% intracochlear
			28.88% transmodiolar
			15.55% intravestibular
			11.11% intravestibulocochlear
			8.88% transmacular
			4.47% transotic
Tieleman et al.	2008	52	80.7% intracochlear
			13.5% intravestibular
			5.8% intravestibulocochlear
Kennedy et al.	2004	28	32% intracochlear
			21% intravestibular
			32% transmodiolar
			11% transmacular
			4% transotic

**Figure 2:**
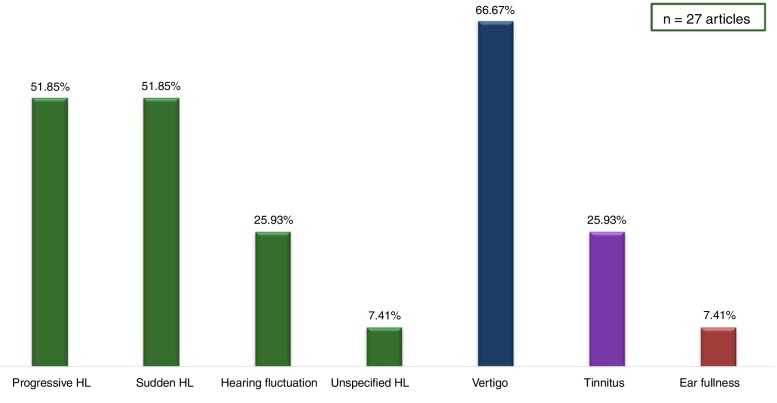
Main manifestations of the intralabyrinthine schwannomas in the 27 articles.

Regarding the patients’ clinical picture, the main clinical manifestations are shown in Table 4 and Fig. 2.

**Table 4:** Main manifestations of intralabyrinthine schwannomas.

Author	Year	*n*	Clinical picture
Plontke et al.	2017	12	100% hearing loss
Covelli et al.	2017	1	Hearing fluctuation and vertigo
Fukushima et al.	2017	1	Sudden hearing loss
Plontke et al.	2017	1	Sudden hearing loss
Sabatino et al.	2017	1	Rapidly progressive hearing loss and vertigo
Jerin et al.	2016	5	40% progressive hearing loss
			40% sudden hearing loss
			20% vertigo
Shupak et al.	2016	7	95% progressive hearing loss
Gosselin et al.	2015	66	No description of the clinical case
Lee et al.	2015	1	Sudden hearing loss and vertigo
Dubernard et al.	2014	110	94.5% progressive hearing loss
			59.1% vertigo
Bittencourt et al.	2014	1	Hearing fluctuation and tinnitus
Kim et al.	2013	1	Sudden hearing loss
Schutt et al.	2013	1	Hearing fluctuation, ear fullness and vertigo
Van Abel et al.	2013	234	84% progressive hearing loss
			3% hearing fluctuation
			43% vertigo
Salzman et al.	2012	45	60% progressive hearing loss
			31.11% sudden hearing loss
			8.89% hearing fluctuation
			35.56% vertigo
Gordts et al.	2011	1	Hearing fluctuation and tinnitus
Magliulo et al.	2009	1	Sudden hearing loss and vertigo
Brozek-Madry et al.	2009	1	Sudden hearing loss and vertigo
Tieleman et al.	2008	52	83.67% progressive hearing loss
			14.28% sudden hearing loss
			19.23% vertigo
Jia et al.	2008	4	75% progressive hearing loss
			25% sudden hearing loss
			75% vertigo
Nishimura et al.	2008	1	Sudden hearing loss and tinnitus
Lella et al.	2007	7	71.42% progressive hearing loss
			28.5% sudden hearing loss
			57.14% vertigo
Kennedy et al.	2004	28	61% progressive hearing loss
			32% sudden hearing loss
			7% hearing fluctuation
			71% tinnitus
			29% vertigo
Green et al.	1999	4	75% progressive hearing loss
			25% sudden hearing loss
			75% vertigo
Deux et al.	1998	3	Progressive hearing loss, tinnitus and vertigo
Weed et al.	1994	1	Progressive hearing loss and tinnitus
De Lozier et al.	1979	2	Progressive hearing loss and vertigo

Patient management reporting was carried out for 486 patients. In 276 patients (56%), clinical follow-up with serial MRI was chosen. In 210 (44%) patients, the patient chose the surgical management since most of them were patients with profound hearing loss and tumor growth evidenced by imaging examination and/or incapacitating vertigo without improvement with clinical treatment. Of the 210 patients submitted to surgical treatment, there are reports of cochlear implant surgery simultaneously or immediately after tumor excision in 11 patients.

### Description of a case of intralabyrinthine schwannoma of the intravestibular subtype according to the modified Kennedy classification^7^

A 38-year-old female patient came to the outpatient clinic to have a consultation with an otorhinolaryngologist due to dizziness and imbalance for 3 years. She reported that each episode lasted only a few seconds, with no triggering factors or symptom improvement or worsening. The events were of minimal intensity and did not hinder the activities of daily living. After a few weeks of the condition onset, the dizziness became rotational and was triggered by head movements to the left. This dizziness symptom with a new characteristic lasted for minutes and also ceased spontaneously. When questioned, she denied headaches, visual or auditory symptoms associated with dizziness. She also denied comorbidities or continuous intake of medications for any disease.

The general physical examination, otorhinolaryngological evaluation and neurological tests did not show any alterations, nor did the auditory assessment (audiometry and immittance audiometry). The patient was also submitted to video-electronystagmography, which showed left hypofunction on the caloric test. Considering the results of the physical, audiometric and vestibular function tests, β-histamine therapy was started, of which dose was gradually increased in the first 60 days until reaching the maximum recommended dose (216 mg/day) in addition to meclizine. However, the patient did not show improvement with the use of the medications.

Four months after the initial consultation, the patient noticed a worsening in the frequency and intensity of rotational dizziness when exposed to specific sounds, such as alarms and sirens; the dizziness ceased as soon as the sound triggering it was interrupted. Six months after the condition onset, she also noticed ear fullness in the left ear associated with a hissing-type tinnitus, in addition to difficulty in understanding sounds in that ear.

**Figure 3:**
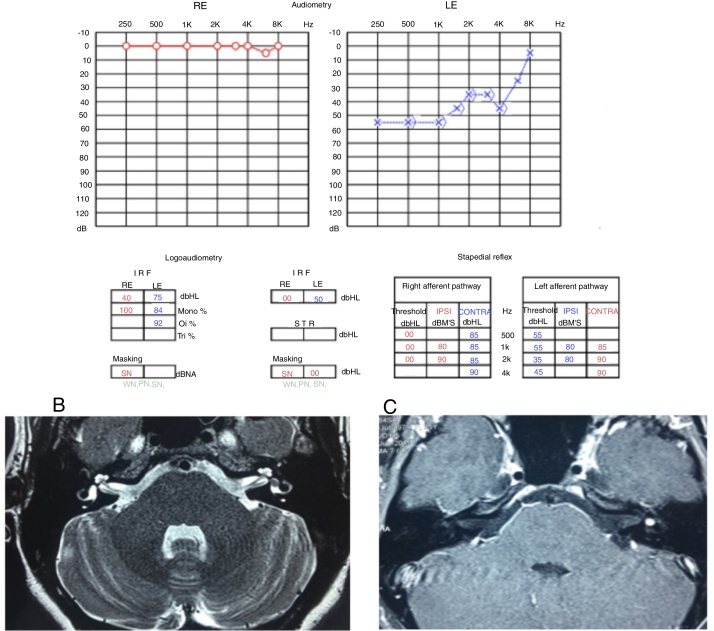
(A) Audiometry showing moderate sensorineural loss in the left ear. (B) T2-weighted MRI of the temporal bones, axial view, with hyposignal in the left vestibule. (C) T1-weighted MRI of the temporal bones, axial view, with contrast, lesion shows hyperuptake in the left vestibule.

These symptoms (ear fullness, tinnitus and alterations of comprehension) showed fluctuation, with moments of worsening and spontaneous improvement; in the moments of hearing worsening, she also noticed rotational dizziness worsening. Considering the worsening of the symptoms, the patient was evaluated by new serial audiometries, which showed fluctuation of auditory thresholds compatible with clinical worsening. She then underwent an MRI of the temporal bones, which disclosed a left vestibular lesion (Fig. 3).

**Figure 4:**
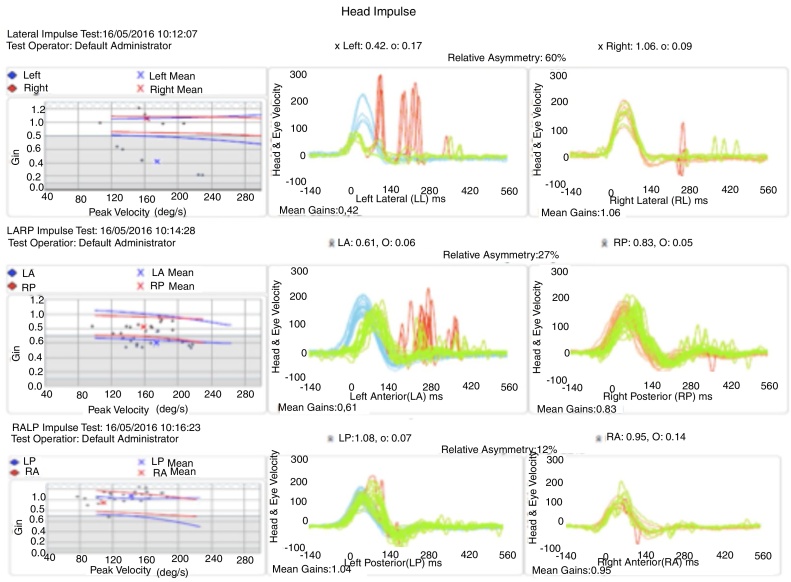
Head-impulse test showing left vestibular hypofunction.

When she underwent vestibular function examination by vestibulo-ocular reflex detection through the had impulse test, she showed a normal vestibular-ocular reflex in all right semicircular canals assessed. On the other hand, regarding the left canals, there was a decreased vestibulo-ocular reflex gain in the lateral and anterior canals, with covered and uncovered corrective saccades; however, the vestibulo-ocular reflex gain of the left posterior canal was normal (Fig. 4). Based on this examination, vestibular hypofunction was identified in the left superior vestibular nerve topography.

**Figure 5:**
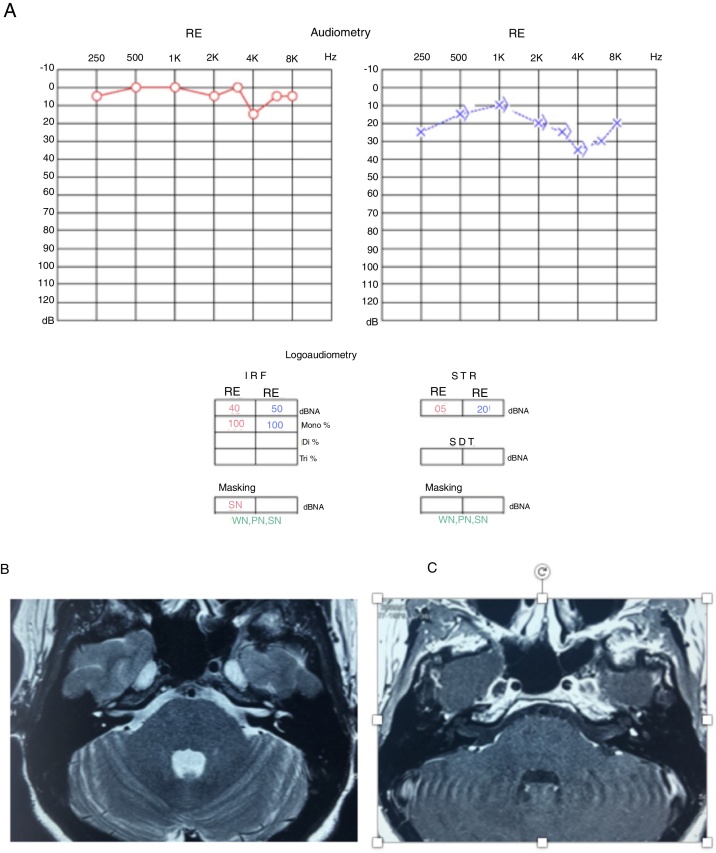
(A) Audiometry showing mild sensorineural loss in the left ear. (B) T2-weighted MRI of temporal bones, axial view, with hyposignal in the left vestibule. (C) T1-weighted MRI of temporal bones, axial view, with contrast, lesion shows hyperuptake in the left vestibule.

Because it was a millimetric lesion, with little impact from the auditory point of view, it was decided to follow the patient by repeating the MRI and the audiometry annually (Fig. 5).

In the third year of follow-up, the patient showed sudden hearing loss in the left ear, without worsening of the vestibular complaints. A new audiometry showed profound hearing loss in the left ear, compatible with the diagnosis of sudden sensorineural hearing loss, or sudden deafness (Fig. 5).

**Figure 6:**
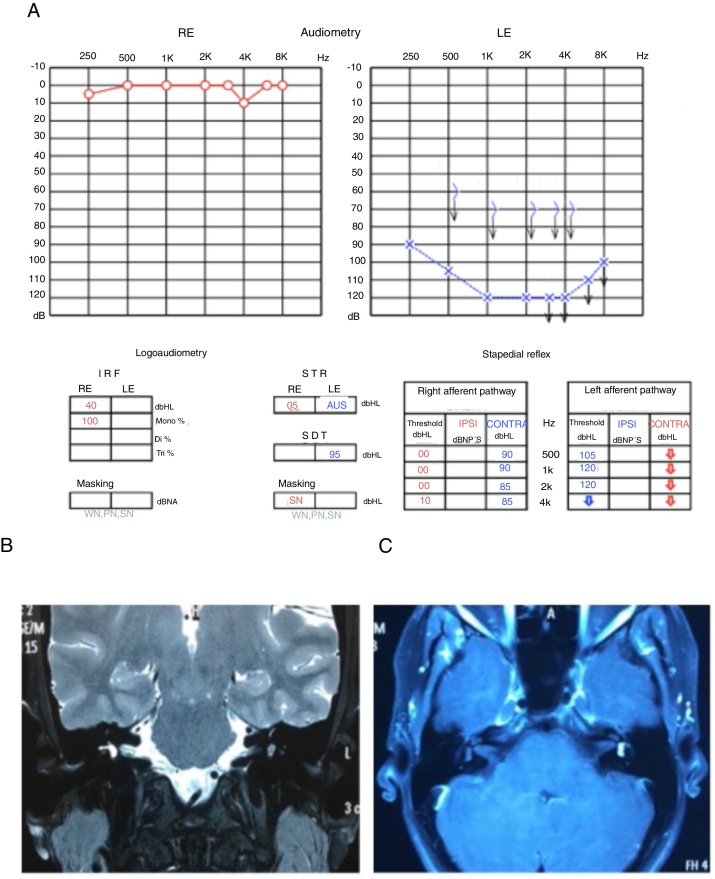
(A) Audiometry showing deep sensorineural loss in the left ear. (B) T2-weighted MRI of temporal bones, coronal view, with hyposignal in the left vestibule. (C) T1-weighted MRI of temporal bones, axial view, T1 with contrast, lesion shows hyperuptake in the left vestibule.

At this time, treatment with oral prednisolone (1 mg/kg/day) was started, lasting for 21 days at regressive doses, and an MRI of the temporal bones was carried out (Fig. 6). The MRI showed tumor growth of approximately 1 mm in comparison to the last examination performed 2 years earlier (Fig. 5). The audiometry showed considerable worsening of auditory thresholds in the left ear (Fig. 6).

**Figure 7:**
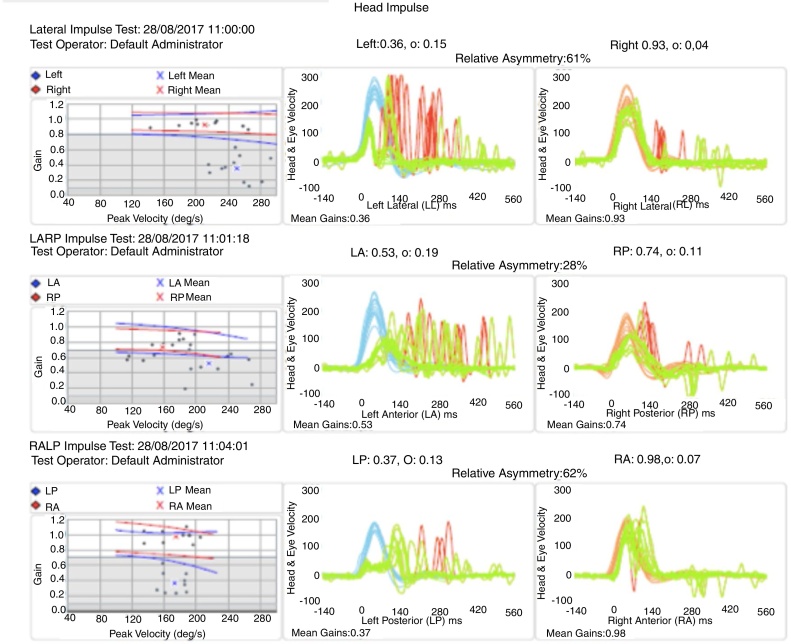
Head-impulse test showing left vestibular hypofunction in all assessed semicircular canals.

Repeating the vestibular function examination by detecting the vestibulo-ocular reflex through the head impulse test at that moment showed that the previously investigated right semicircular canals remained normal. There was a significant decrease in gain at the left in all assessed semicircular canals with compensatory saccades (Fig. 7).

After 21 days of treatment, the patient did not show any improvement from the point of view of hearing function or body balance. Therefore, translabyrinthine surgery was chosen, as the patient had no hearing to be preserved as she had before.

## Discussion

It is still difficult to estimate the real prevalence of intralabyrinthine schwannomas, due to the small number of reported cases and the lack of specificity of their clinical manifestations, which makes them difficult to diagnose. Moreover, the clinical picture may suggest other more frequent diagnoses, which include Meniere's disease, vestibular neuritis or sudden idiopathic hearing loss.^10^ In our review, we found a total of 526 reported cases of intralabyrinthine schwannoma. In relation to the most frequent subtype, meta-analyses and systematic reviews suggest that the most commonly found subtype is the intracochlear, followed by the intravestibular and intravestibulocochlear types (Table 3).

Another topic still debated in several studies is the tumor origin. Although some studies suggest that it is not possible to clearly identify the tumor origin,^11^ Neff et al.,^12^ in a review of 55 cases (23 intracochlear, 25 intravestibular and 7 involving the cochlea and vestibule), concluded that there seems to be no difference between the incidence of tumor genesis in the cochlear nerve or vestibular nerve.

As for the clinical picture of the patients with intralabyrinthine schwannoma, there have been no studies that correlated tumor location with patient complaint. Moreover, studies describing long-term follow-up, changes in the clinical picture during tumor evolution and tumor radiological evolution are scarce and document limited samples. Therefore, our study seems to be the first one in the literature to correlate, based on a case report, the tumor subtype with the clinical manifestations, in addition to describing the clinical evolution and results of auditory and imaging tests in a long-term follow-up.

In our review, the main clinical manifestation observed in patients with intralabyrinthine schwannoma, regardless of the subtype, is hearing loss, being usually sensorineural, progressive, with low speech discrimination.^12^ It is estimated that 15%–32% of patients have sudden hearing loss as the initial manifestation of the condition, with rare cases of hearing loss fluctuation.^13^, ^14^ The second most frequent complaint is vertigo, which may occur secondary to all intralabyrinthine schwannoma subtypes.

Although there is still no consensus regarding the pathophysiology of hearing loss in these cases, it is believed that in the intracochlear schwannoma, the loss is a consequence of direct compression or destruction of the cochlear nerve. In tumors located inside the vestibule, the hearing loss can be explained by the hydrolymphatic hydrops, which would cause compression of the adjacent structures.^15^ In both cases, the tumor can also cause metabolic alterations in the inner ear fluids, leading to hearing loss and imbalance.^16^

Although not limited to it, the complaint of vertigo occurs more frequently in the intravestibular schwannoma.^17^, ^18^ Regarding the treatment of intralabyrinthine schwannomas, in most of the cases reported in the selected studies, clinical and serial radiological follow-up was chosen, considering the low percentage (15%) of patients that showed tumor growth within a 5-year evolution period.^18^ In some situations, it is possible to choose surgical excision of the tumor, with the main indications being (1) evidence of tumor growth leading to involvement of the cerebellopontine angle, internal auditory meatus or middle ear; (2) total hearing loss; (3) vestibular symptoms that do not improve with clinical treatment; or (4) diagnostic doubt.^1^ A review published by Van Abel et al.^2^ including 14 patients diagnosed with labyrinthine schwannoma who underwent clinical follow-up, estimated that only 3% required surgical treatment. Considering the high risk of complications during the surgery for the labyrinthine schwannoma removal (including deafness, dizziness and facial paralysis) and the low growth potential of these tumors, studies suggest that the clinical follow-up is best, with surgery being reserved for specific situations or signs of untreatable symptoms.

## Conclusion

The main clinical manifestation of patients with intralabyrinthine schwannoma is hearing loss, present in approximately 100% of the reported cases.^19^, ^20^ The most frequent type of hearing loss is the sensorineural type, which is characteristically progressive; sudden or fluctuating losses are less frequent. Although there are almost 600 cases of intralabyrinthine schwannoma reported in the literature, detailed descriptions of the patients’ clinical evolution and the corresponding radiological correlations are scarce. The embryological and pathophysiological mechanisms involved in the genesis of the tumor and its consequent auditory sequelae are yet to be completely elucidated.

## Conflicts of interest

The authors declare no conflicts of interest.
